# The Role of Ferroptosis in Cancer Development and Treatment Response

**DOI:** 10.3389/fphar.2017.00992

**Published:** 2018-01-12

**Authors:** Bin Lu, Xiao Bing Chen, Mei Dan Ying, Qiao Jun He, Ji Cao, Bo Yang

**Affiliations:** Zhejiang Province Key Laboratory of Anti-Cancer Drug Research, College of Pharmaceutical Sciences, Zhejiang University, Hangzhou, China

**Keywords:** ferroptosis, lipid ROS, iron metabolism, cancer therapeutics, tumorigenesis

## Abstract

Ferroptosis is a process driven by accumulated iron-dependent lipid ROS that leads to cell death, which is a distinct regulated cell death comparing to other cell death. The lethal metabolic imbalance resulted from GSH depletion or inactivation of glutathione peroxidase 4 is the executor of ferroptosis within the cancer cell. Small molecules-induced ferroptosis has a strong inhibition of tumor growth and enhances the sensitivity of chemotherapeutic drugs, especially in the condition of drug resistance. These evidences have highlighted the importance of ferroptosis in cancer therapeutics, but the roles of ferroptosis in tumorigenesis and development remain unclear. This article provides an overview of the mechanisms of ferroptosis, highlights the role of ferroptosis in cancer and discusses strategies for therapeutic modulation.

## Introduction

Ferroptosis, identified in 2012 by Dr. Brent R. Stockwell, is an iron-dependent form of non-apoptotic regulated cell death (Dixon et al., 2012). Ferroptosis is morphologically, biochemically, and genetically distinct from other well-known forms of cell death, including apoptosis, various forms of necrosis, and autophagy (Yagoda et al., 2007; Yang and Stockwell, 2008; Dixon et al., 2012; Fatokun et al., 2014). Apoptosis is a classical regulated cell death executed by dedicated pathways involving key pro-death effector proteins such as BCL2-associated X protein (BAX). However, ferroptosis is initialized through GSH depletion or inactivation of GPX4 activity (Dixon et al., 2012, 2014; Yang et al., 2014). During the ferroptosis, canonical hallmarks of apoptosis were not observed in several cancer cells, such as poly (ADP ribose) polymerase 1 (PARP1) cleavage, cytochrome c releasing from mitochondria, or pro-caspase-3 cleavage (Yagoda et al., 2007; Yang and Stockwell, 2008). Besides, there are special and distinct changes in mitochondrial morphology during the ferroptosis (Xie et al., 2016), mainly the loss of structural integrity, such as smaller than normal mitochondria with condensed mitochondrial membrane densities, reduced or absent mitochondria crista (Yagoda et al., 2007; Dixon et al., 2012) and outer mitochondrial membrane rupture (Friedmann Angeli et al., 2014). Thus, there are no cross-talk in the initial mechanisms or downstream evens between apoptosis and ferroptosis. So far, several groups have demonstrated that, in the early stage of ferroptosis, autophagy contributes to ferroptosis through providing available labile iron via NCOA4-mediated ferritinophagy (Gao et al., 2016; Hou et al., 2016). However, once ferroptosis is induced, especially in the later upstream events, autophagy maybe no longer a key regulator.

The overwhelming iron-dependent accumulation of lipid peroxidation products is considered the main killer in the ferroptotic process. Thus, small-molecule lipophilic antioxidants (e.g., ferrostatin-1 and α-tocopherol) and iron chelators (e.g., deferoxamine) can prevent ferroptotic cell death, whereas inhibitors of the other abovementioned forms of cell death cannot accomplish this feat. It is well-established that system Xc^-^ (Dixon et al., 2014) and GPX4 (Yang et al., 2014) are key regulators of ferroptosis. Two classical compounds, erastin (Dolma et al., 2003) and (1S, 3R)-RSL3, can induce ferroptosis through the inhibition of system Xc^-^ and GPX4, respectively. Cell death is crucial for normal development, homeostasis, and prevention of hyperproliferative diseases, such as cancer. Cancer cells can undergo several forms of regulated cell death during tumor development (e.g., apoptosis), and activation of regulated cell death is a primary and promising strategy for cancer therapy. Despite success in clinical cancer treatments, drug resistance to existing chemotherapeutic agents due to genetic alterations remains a problem. Intriguingly, erastin, a ferroptosis inducer, shows the ability to enhance the effectiveness of chemotherapy drugs (e.g., temozolomide, cisplatin, cytarabine/ara-C, and doxorubicin/Adriamycin) in certain cancer cells (Chen et al., 2015; Yu et al., 2015; Roh et al., 2016). Notably, RCCs, B cell-derived lymphomas and a subset of triple-negative breast cancer cell lines acquire a strong dependence on GPX4 and system Xc^-^ (Conrad et al., 2016). These results indicate that inducing ferroptosis maybe a new therapeutic anticancer strategy in tumors, especially tumors containing the two-abovementioned metabolic signatures. p53 is a crucial tumor suppressor and is mutant in most cancer cells. Recently, the tumor-suppressive activity of p53 was partially attributed to its ability to induce ferroptosis via inhibiting system Xc^-^ (Jiang et al., 2015); thus, ferroptosis may play a crucial role in tumorigenesis. In addition, some clinical drugs approved by the FDA [e.g., sorafenib (Dixon et al., 2014), sulfasalazine and ART] can also induce ferroptosis in several cancer cells, supporting the feasibility of using ferroptosis in preclinical and clinical settings. Taken together, ferroptosis shows an intimate association with tumors and cancer therapy, although it’s exact function in cancer remains unelucidated. In this review, we discuss the role of ferroptosis in tumorigenesis and cancer therapy, providing an improved understanding of ferroptotic mechanism-based therapeutic approaches.

## Mechanisms of Ferroptosis

**FIGURE 1:**
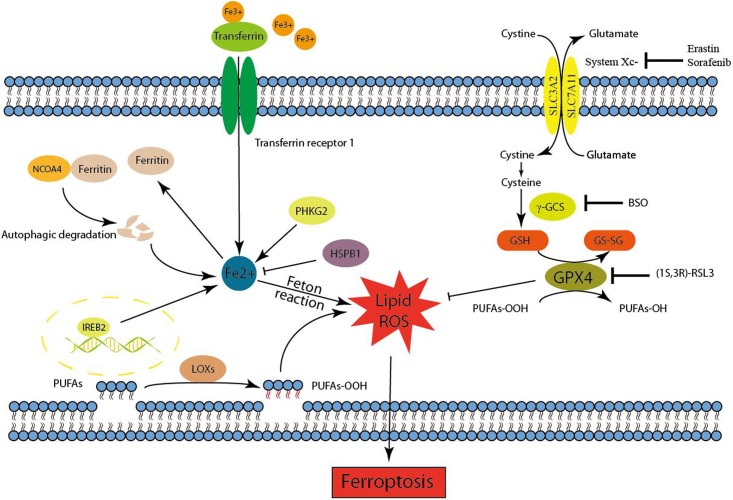
Mechanism of ferroptotic cell death. Ferroptosis is initiated by the inhibition of system Xc^-^ or GPX4 activity, which ultimately leads to cell death. Lipid ROS maybe responsible for the ferroptotic process. On the one hand, the peroxidation of PUFAs is considered to be an important contributor. On the other hand, excess irons are the basis for ferroptosis execution.

Ferroptosis is initiated by the loss of GPX4 activity, which is mediated by two distinct mechanisms (**Figure 1**). The first mechanism is the inhibition of system Xc^-^ (e.g., mediated by erastin), which indirectly inhibits GPX4 (Dixon et al., 2012, 2014). System Xc^-^ is a cystine/glutamate antiporter that imports extracellular cystine in exchange for intracellular glutamate (Bridges et al., 2012). Cysteine, a reduced form of cystine, is a precursor for the synthesis of GSH. By using GSH as an essential cofactor, GPX4 exerts its phospholipid peroxidase activity to catalyze the reduction of lipid peroxides. Thus, the inhibition of system Xc^-^ by small molecules causes GSH depletion and subsequently inactivates GPX4, ultimately leading to the accumulation of lethal lipid peroxides and the initiation of ferroptosis (Dixon et al., 2012, 2014; Yang et al., 2014). Notably, inhibition of γ-GCS (e.g., mediated by buthionine sulfoximine), which is the rate-limiting enzyme for the synthesis of GSH, is also sufficient to initiate ferroptosis (Yang et al., 2014). Another molecular mechanism of ferroptosis is the direct inhibition of GPX4 through the loss of its activity or promoting its degradation. The classical ferroptotic inducer (1S, 3R)-RSL3, can inhibit GPX4 enzymatic activity by covalently targeting its active site selenocysteine in an irreversible manner (Yang et al., 2014, 2016). FIN56, a new and specific ferrotosis inducer, is involved in decreasing GPX4 abundance. However, the detailed mechanism remains unclear, maybe related to the enzymatic activity of acetyl-CoA carboxylase (Shimada et al., 2016). Furthermore, genetic inhibition of GPX4 by siRNA was also sufficient to induce the accumulation of lipid ROS and ferroptotic cell death (Yang et al., 2014). In general, GPX4 is a central regulator of ferroptosis-triggering mechanisms. Given that the iron-dependent accumulation of lipid ROS kills cells undergoing ferroptosis, both iron metabolism and lipid peroxidation are two critical processes involved in the mechanism of ferroptosis. Therefore, the following contexts were mainly carried out in these two aspects (**Figure 1**).

### Iron Metabolism

Redox-active iron pools (i.e., Fe^2+^) are capable of directly catalyzing lipid peroxides to form damaging free radical via Fenton chemistry (**Figure 1**). Once lipid peroxides are not removed in time within the cell, constantly accumulative lipid alkoxy (RO ⋅) radicals will lead to ferroptosis ultimately. But this is just one of the reasons to explain why ferroptosis is dependent on iron rather than other metal ions.

#### Iron Metabolism Genes

Iron response element binding protein 2 (IREB2) has been identified as an essential gene for erastin-induced ferroptosis in HT-1080 and Calu-1 cells (Dixon et al., 2012). IREB2, also named IRP2, plays a crucial role in controlling intracellular and systemic iron homeostasis by post-transcriptionally controlling iron metabolism genes via the IRE–IRP system. These iron metabolism genes comprise ferritin [*FTH1*, *FTL*, *TF*, *TfR1*, *Fpn*, and *DMT1* (Bogdan et al., 2016)]. Ferritin is the major intracellular iron storage protein, and the NCOA4-mediated autophagic degradation of ferritin (namely ferritinophagy) contributes to erastin-induced ferroptosis (Hou et al., 2016). Genetic inhibition of ATG5, ATG7, or NCOA4 decreased ferroptotic cell death, which was accompanied by decreased intracellular ferrous iron levels and lipid peroxidation end products (e.g., MDA). Moreover, the inhibition of lysosomal activity also decreased ferroptosis. Transferrin (TF), an iron-binding serum protein, can be transported into the cell via TfR1-mediated endocytosis (Bogdan et al., 2016). TF was identified as an executioner of serum-dependent cell death upon cystine starvation. Further experiments demonstrated that TF is an essential component for the induction of ferroptotic cell death. In addition, only TF transported into the cell with iron loaded by TfR1 can exert its corresponding ability (i.e., inducing ferroptosis) in certain contexts (Gao et al., 2015). In general, a certain amount of available labile iron is the basis for ferroptosis execution.

#### Other Regulators

Heat shock protein family B (small) member 1 (HSPB1), a small heat shock protein, is a negative regulator of erastin-induced ferroptosis *in vitro* and *in vivo* (Sun et al., 2015). HSPB1 can downregulate TfR1-mediated iron uptake by stabilizing actin cytoskeleton, and HSPB1 overexpression decelerates transferrin endocytosis and recycling (Chen et al., 2006).

Heme oxygenase 1 (HO-1) plays a dual role in the regulation of ferroptosis. Increased expression of HO-1 is an important event in erastin-induced ferroptosis in HT-1080 fibrosarcoma cells, and HO-1 likely provides iron supplements for promoting ferroptosis (Kwon et al., 2015). Intriguingly, another study suggested that HO-1 negatively regulated erastin- or sorafenib-induced ferroptosis in HCC cells (Sun et al., 2016). HO-1 knockdown by shRNA enhanced growth inhibition in response to erastin and sorafenib in HCC cells. The role of HO-1 in ferroptosis maybe dependent on different pathological contexts, although the precise mechanism of this phenomenon needs to be studied further.

Phosphorylase kinase catalytic subunit gamma 2 (PHKG2) positively regulates ferroptosis through the modulation of available iron, and PHKG2 silencing may function as iron chelation (Yang et al., 2016). The detailed mechanism of PHKG2 in iron metabolism regulation is unknown and needs further investigation.

### Lipid Peroxidation

The overwhelming accumulation of lipid ROS is the ferroptosis executioner, which can be prevented by lipophilic antioxidants and iron chelators. NOXs provide a source of accumulated ROS in erastin-induced ferroptosis (Dixon et al., 2012). Indeed, the pharmacological inhibition of NOXs and the NADPH-generating PPP strongly rescued erastin-induced ferroptosis in Calu-1 cells. However, pharmacological inhibition of NOXs or PPP partially rescued erastin-induced ferroptosis in HT-1080 cells, especially loss of inhibition in high concentration of erastin. The contradictory results may due to different cell lines, reflecting that NOX/PPP pathway is likely to be a downstream consequence rather than an initiation factor. In addition to NOXs, cell membrane lipid peroxidation products are another source of ROS generation, which really drives ferroptosis execution.

In membrane lipid environments, PUFAs, but not monounsaturated FA, cholesterol, and cardiolipin, are specifically peroxidized in ferroptosis (Yang et al., 2016). Indeed, PUFAs [e.g., arachidonic acid (AA, 20:4n6)] and PUFA derivatives [e.g., linoleate (18:2n6)] were significantly decreased following erastin treatment in HT-1080 cells (Skouta et al., 2014). Numerous regulators and pathways involving the synthesis of FA, such as glutaminolysis (Gao et al., 2015), citrate synthase (Dixon et al., 2012), and acetyl-CoA carboxylases, are necessary for the execution of ferroptosis (Shimada et al., 2016). ACSL4 preferentially acylates AA, while LPCAT3 preferentially catalyzes the insertion of acylated AA into membrane phospholipids, ultimately leading to the conversion of lysoPC to PC (Dixon et al., 2015). These two genes are essential for the execution of ferroptosis induced by the inhibition of GPX4. Moreover, ACSF2 is also required for erastin-induced ferroptosis (Dixon et al., 2012). Taken together, these genes ensure adequate membrane lipid PUFA production to promote ferroptosis for subsequent lipid peroxidation and ROS generation.

Lipoxygenases can drive ferroptosis, mediated by system Xc^-^ inhibition, by catalyzing the dioxygenation of membrane lipid PUFAs to produce fatty acid hydroperoxides (Yang et al., 2016). For example, Zileuton (Rossi et al., 2010), a 5-lipoxygenase inhibitor, confers neuroprotection against glutamate oxidative damage by inhibiting ferroptosis (Liu et al., 2015). Under normal conditions, fatty acid hydroperoxides are converted to fatty acid alcohols under GPX4 mediation. However, this process is blocked during ferroptosis because of GPX4 inactivation. Accumulated fatty acid hydroperoxides are further catalyzed into lipid peroxyl radicals by iron-mediated Fenton reactions, which are lethal to cells. The oxidative destruction of membrane lipid PUFAs increases with ferroptosis, as lysoPC accumulates and specific PUFAs (Skouta et al., 2014). A study showed that resistance to system Xc^-^ inhibition dramatically increased the expression of AKR1C (Dixon et al., 2014), which can detoxify cytotoxic oxidative PUFA breakdown products (e.g., MDA and 4-HNE). However, the detailed mechanism underlying the direct inhibition of GPX4-induced ferroptosis is still enigmatic.

## Ferroptosis and Tumor Suppression

Cancer cells can undergo several regulated forms of cell death during tumor development, including apoptosis (Kerr et al., 1994), autophagy (Kondo et al., 2005; Levine, 2006), and necrosis (Amaravadi and Thompson, 2007). Unsurprisingly, ferroptosis also plays a role in the development of cancer and maybe a beneficial strategy for anticancer treatment.

Different lines of evidence suggest that ferroptosis plays a crucial role in the suppression of tumorigenesis. Specifically, GPX4 knockdown using siRNAs decreases the level of GPX4 protein, a central mediator of ferroptosis, inducing renal cell carcinoma cell death with accompanying lipid ROS generation (Yang et al., 2014). This process can be rescued by the iron chelator DFO and the antioxidant vitamin E. Moreover, inhibition of a member of cystine/glutamate antiporter, SLC7A11, can induce ferroptosis (Dixon et al., 2012). SLC7A11 is highly expressed in human tumors (Huang et al., 2005; Liu X. X. et al., 2011), and its overexpression rescues cancer cells from ferroptosis. Both the pharmacological and genetic inhibition of SLC7A11 induce ferroptotic cell death and enhance cisplatin cytotoxicity in cisplatin-resistant head and neck cancer (HNC) cells *in vitro* and *in vivo* (Roh et al., 2016). In addition, a recent study showed that p53 inhibits tumor growth partially by repressing the expression of SLC7A11 and subsequently inducing ferroptosis in addition to traditional p53-mediated functions, such as cell cycle arrest, senescence, and apoptosis (Jiang et al., 2015). Notably, p53^3KR^, an acetylation-defective mutant that fails to induce cell cycle arrest, senescence and apoptosis, fully retains its ability to regulate SLC7A11 expression and induce ferroptosis upon ROS-induced stress (Jiang et al., 2015). p53^3KR^ mice did not succumb to the early-onset spontaneous thymic lymphoma formation that is commonly observed in p53-null mice (Li et al., 2012). Consistently, unlike p53^-/-^XRCC4^-/-^ mice, p53^3KR/3KR^XRCC4^-/-^ mice did not succumb to pro-B cell lymphomas (Li et al., 2016). This suggests that p53^3KR^ retains its tumor-suppressive activity and that uncanonical functions of p53 play crucial roles in the suppression of tumorigenesis. Taken together, these findings indicate that ferroptosis serves as a critical barrier to cancer development.

Accumulating evidence has shown that the activation of ferroptosis contributes to anticancer treatment for several forms of human cancer (**Table 1**). Erastin, a RAS-selective lethal compound (Dolma et al., 2003) that triggers ferroptosis by directly inhibiting cystine/glutamate antiporter system Xc^-^ activity, displays synthetic lethality in engineered cells. However, erastin does not show selective lethality in RAS-mutated cancer cell lines over RAS wild-type counterparts in a large, diverse panel of cell lines. Analysis of erastin sensitivity data from 117 cell lines and a 60-cancer cell line panel (NCI 60) harvested from eight diverse tissues (leukemia, lung, colon, CNS, melanoma, ovarian, kidney, and breast) revealed that diffuse, large B cell lymphomas and RCCs are particularly sensitive to ferroptosis (Yang et al., 2014). Moreover, low-cytotoxic doses of erastin remarkably enhance the anticancer activities of 2 first-line chemotherapy drugs, cytarabine and doxorubicin, in HL60 cells (Yu et al., 2015). Given that AML has a high recurrence rate, inducing ferroptosis may overcome drug resistance in AML cells.

**Table 1:** Ferroptosis relevance among various types of cancer.

Cancer type	Relevance to ferroptosis	Test animals
Breast	Combined treatment of siramesine and lapatinib induces ferroptosis by upregulating iron levels (Ma et al., 2016)	None
Head and neck	Induction of ferroptosis makes cisplatin-resistant cells more sensitive to treatment (Roh et al., 2016); dihydroartemisinin-induced cell death is partially involved in ferroptosis (Lin et al., 2016)	Nude mice/none
Acute myeloid leukemia	Erastin enhances the sensitivity of AML cells to cytarabine and doxorubicin (Yu et al., 2015)	None
Pancreatic ductal adenocarcinoma	Artesunate (ART) induces cell death through ferroptosis dependent upon antioxidant homeostasis and increased sensitivity to free intracellular iron (Eling et al., 2015)	None
Ovarian	The antiproliferative and cytotoxic effects of ART are partially due to ferroptosis (Greenshields et al., 2016)	C57BL/6 mice
Hepatocellular carcinoma	The cytotoxic effects of sorafenib involves ferroptosis; inhibition of the p62-Keap1-NRF2 pathway increases the anticancer activities of erastin and sorafenib; sorafenib-resistant cells are more sensitive to ferroptosis (Galmiche et al., 2014; Louandre et al., 2015; Sun et al., 2016)	Nude mice/C57BL/6 mice
Cervical carcinoma Osteosarcoma Prostate adenocarcinoma	HSPB1 is highly inducible following erastin treatment (Sun et al., 2015)	NOD/SCID mice
Diffuse large B cell lymphoma	Particularly sensitive to ferroptotic cell death; dependent on system Xc^-^ (Yang et al., 2014)	None/none
Renal cell carcinomas	More sensitive to ferroptosis compared with seven other tissues (Yang et al., 2014)	
Non-small cell lung	Erastin enhances the effect of cisplatin in NSCLCs (Yamaguchi et al., 2013)	Nude mice
Glioblastoma	xCT and cystathionine γ-lyase are inducible after temozolomide treatment; erastin enhances sensitivity to temozolomide (Chen et al., 2015; Sehm et al., 2016)	None/none
Triple-negative breast	Targeting the MUC1-C/xCT pathway could be a therapeutic approach (Hasegawa et al., 2016)	None

Sorafenib, a multikinase inhibitor, is clinically approved for the treatment of renal cell carcinoma and other indications. Recently, sorafenib was identified as a ferroptosis inducer by inhibiting system Xc^-^ function and triggering subsequent ferroptotic cell death (Dixon et al., 2014). However, acquired resistance to sorafenib has been found in HCC patients, which results in poor prognosis. Previous studies show that NRF2 (Sun et al., 2016) and MT-1G (Houessinon et al., 2016), two negative regulators of ferroptosis that function by blocking GSH depletion-mediated lipid peroxidation in HCC cells, play a central role in protecting HCC cells against ferroptosis and result in sorafenib resistance. Importantly, the genetic and pharmacological inhibition of NRF2 or MT-1G in HCC cells enhances the anticancer activity of sorafenib *in vitro* and in tumor xenograft models.

In addition, numerous molecules, such as RSL3, RSL5, ART (Eling et al., 2015), DHA (Lin et al., 2016), and a series of small-molecule inducers (FINs) (Weiwer et al., 2012; Yang et al., 2012), have been shown to introduce ferroptotic cell death in cancer cells. Co-treatment with the ferroptosis inhibitor ferrostatin-1 can block ART-induced lipid peroxidation and cell death and increase long-term cell survival and proliferation.

As discussed above, ferroptosis participates in cancer development and treatment response, contributing to tumor suppression activity. Although many experiments *in vitro* have shown that ferroptosis can be detected via several measuring methods, such as cell viability, iron level and ROS level, it would be harder to prove the presence of ferroptosis *in vivo*. So far, *PTGS2*, a gene encoding cyclooxygenase-2 (COX-2), is the unique widely used marker for erastin or RSL-3-induced ferroptosis in tumor (Yang et al., 2014).

## Therapeutic Targeting of Ferroptosis Activation

Activation of regulated cell death is an anticancer treatment strategy. Despite success in clinical cancer treatment, drug resistance to existing chemotherapeutic agents due to genetic alterations remains a problem (Pommier et al., 2004; Liu L. et al., 2011; Sui et al., 2013). Considering the clinical limitations and the discovery of ferroptosis (Dixon et al., 2012), activation of a regulated non-apoptotic form of cell death, ferroptosis, may provide new drug targets.

As described above, compelling evidence suggests that the activation of ferroptosis is an effective, novel pathway for cancer intervention. In principle, ferroptosis activation could be achieved by (i) the identification of small molecules as ferroptosis inducers using systematic screening and structural basis or by (ii) the inhibition of key molecules related to ferroptosis, such as the cystine/glutamate antiporter SLC7A11. We will comment here on the avenues in which progress can be made in cancer treatment by ferroptosis activation.

### Small-Molecule Ferroptosis Activation

Since ferroptosis was identified as a novel form of iron-dependent non-apoptotic cell death, it has been recognized as a new target of drug discovery in recent years. Using systematic screening, a number of small molecules, such as erastin (Dixon et al., 2012), sorafenib (Sun et al., 2016), ART (Eling et al., 2015), DHA (Lin et al., 2016), RSL3, and FINs, were found to induce cell death via ferroptosis. As described above in Section “Ferroptosis and Tumor Suppression,” the combination of erastin and sorafenib maybe a promising therapeutic strategy for cancer, especially in sorafenib-resistant contexts.

In addition, ART specifically induced ROS- and lysosomal iron-dependent cell death in PDAC cell lines (Eling et al., 2015). Co-treatment with the ferroptosis inhibitor ferrostatin-1 blocked ART-induced lipid peroxidation and cell death and increased long-term cell survival and proliferation. DHA specifically causes HNC cell death via contribution from both ferroptosis and apoptosis (Lin et al., 2016). Moreover, 10 different artemisinin derivatives were found to kill tumor cells not only by the induction of apoptosis, autophagy or necroptosis, as shown in the past, but also by ferroptosis (Ooko et al., 2015). In a larger screening to find ferroptosis-inducing compounds, a series of small-molecule inducers, named FINs, were discovered. FIN56 treatment resulted in loss of the GPX4 protein through post-translational degradation and blocked the mevalonate-derived production of lipophilic antioxidants, such as coenzyme Q10 (Shimada et al., 2016). However, the full therapeutic potential of this perspective needs to be explored in more detail in the future.

Given that (1) GPX4 is responsible for ferroptotic cell death induced by lipid peroxidation signaling and (2) knockdown of GPX4 expression using siRNA reagents is sufficient to induce ferroptosis (Yang et al., 2014), GPX4 inhibitors maybe potential candidates for cancer therapy. Considering the success of inhibiting GPX4 by RSL3, finding the structural basis underlying the RSL3 inhibition of GPX4 to design GPX4 inhibitors is a hot research topic. A recent study identified a ligand-binding site on GPX4, and covalently targeting the active site selenocysteine can inhibit GPX4 to induce ferroptosis (Yang et al., 2016). Ideally, a cocrystal structure of RSL3 bound to GPX4 would initiate a search for drug-like GPX4 inhibitors. Systematic screening for ferroptosis inducers and understanding the structural basis for interfering with the active site selenocysteine of GPX4 may confront tumor chemotherapy resistance. Meanwhile, we should consider that GPX4 is an essential enzyme for life and knockout of GPX4 in mice caused embryonic lethality between E7.5 and E8.5 (Yant et al., 2003). But it still could be true that cancer cells will be more sensitive to the inhibition of GPX4 comparing to the normal cells. Thus, the inhibition GPX4 still could be the potential therapeutic approach but the side effects caused by the loss of GPX4 should also be considered in future study.

So far researches on the safety or efficiency of ferroptotic drugs *in vivo* are limited, mainly in piperazine erastin (PE), (1S, 3R)-RSL3, and sorafenib. PE is an analog of erastin with better water solubility and metabolic stability, which is suitable for *in vivo* experiments (Yang et al., 2014). It has been demonstrated that ferroptosis induced by PE or (1S, 3R)-RSL3 prevents tumor growth in xenograft mouse tumor models. Besides, when loss of the retinoblastoma protein, sorafenib has a powerful effect on tumor regression in murine xenografts of HCC (Louandre et al., 2015). However, the above studies have not yet been involved in the evaluation of drug safety. A resolution of this matter will be required in future.

### Interference with Key Ferroptosis Molecules

In addition to GPX4, inhibition of an increasing number of key molecules has been related to ferroptosis, which may induce cell death and eradicate chemotherapy/radiotherapy-resistant cancer cells.

Since the status of NRF2 is a key factor determining the therapeutic response to ferroptosis-targeted therapies in HCC cells (Sun et al., 2016), inhibiting the expression of NRF2 during ferroptosis-targeted therapies to enhance curative tumor effects is necessary. In addition, iron-enriched tumor environments as well as the use of sorafenib to treat RCCs in the clinic suggest that RCCs, especially chromophobe RCCs, could be targeted for ferroptosis induction (Kroll et al., 1984; Sato et al., 2005). A recent study demonstrated that autophagy contributes to ferroptosis by the degradation of ferritin in fibroblasts and cancer cells. Remarkably, NCOA4 was a cargo receptor for the selective autophagic turnover of ferritin (ferritinophagy) in ferroptosis (Hou et al., 2016). Consistently, overexpression of NCOA4 increased ferritin degradation and promoted ferroptosis.

Moreover, several studies have shown that pharmacological and genetic inhibition of the cystine/glutamate antiporter SLC7A11 significantly sensitized resistant HNC cells to cisplatin *in vitro* and *in vivo*, and SLC7A11-deficient mice developed normally and were healthy (Sato et al., 2005), suggesting that compounds targeting system Xc^-^ with high specificity may have minimal side effects in preclinical and clinical settings. However, system Xc^-^ is probably not an effective target for ferroptosis in some cancer, especially cancer cells can bypass their dependence on system Xc^-^. For example, when loss of cysteinyl-tRNA synthetase resulted in the accumulation of cystathionine, the transsulfuration pathway was activated and ultimately led to resistant to erastin-induced ferroptosis (Hayano et al., 2016). In addition, inhibition of the transsulfuration pathway resensitized cells to erastin. On another hand, system Xc^-^ is also a promising target for some cancers, which depend on cysteine/cystine import system (system Xc^-^) due to defective transsulfuration pathway. For example, diffuse large B cell lymphoma were particularly sensitive to erastin-induced ferroptosis (Yang et al., 2014). Thus, whether system Xc^-^ can become a therapeutic target of ferroptosis should be considered the genetic composition of cancer cells.

Collectively, inhibition of key ferroptotic molecules is a promising therapeutic strategy for the treatment of tumor, especially drug-resistant tumors.

## Perspective

The data described herein clearly show that ferroptosis is an iron-dependent form of non-apoptotic cell death and an ideal target for biochemical studies. ROS-induced ferroptosis contributes to tumor growth suppression and chemotherapy sensitivity. Because the activation of ferroptosis is widely recognized as a new target of drug discovery, an increasing number of small molecules have been identified to induce ferroptosis directly or indirectly by targeting iron metabolism and lipid peroxidation, and other studies have focused on the inhibition of key molecules related to ferroptosis. Meanwhile, whether compounds targeting ferroptotic cell death regulators have high specificity and minimal side effects in preclinical and clinical settings and what kinds of cancer prefer to be targeted by ferroptosis induction remain to be elucidated.

In summary, an improved understanding of the ferroptosis mechanism and the role of ferroptosis in cancer will create new opportunities for diagnosis and therapeutic intervention.

## Author Contributions

BY and JC conceived, designed the conception of review article, and made the amendments of the paper. BL conducted the paper. XC, MY, and QH collected the related research articles.

## Conflict of Interest Statement

The authors declare that the research was conducted in the absence of any commercial or financial relationships that could be construed as a potential conflict of interest. The reviewer RP and handling editor declared their shared affiliation.

## Abbreviations

AA: arachidonic acid
ACSF2: acyl-CoA synthetase family member 2
ACSL4: acyl-CoA synthetase long-chain family member 4
AKR1C: aldo–keto reductase family 1 member C
AML: acute myeloid leukemia
ART: artesunate
ATG5: autophagy related 5
ATG7: autophagy related 7
DFO: desferrioxamine
DHA: dihydroartemisinin
DMT1: divalent metal transporter 1
FA: fatty acids
FDA: Food and Drug Administration
FINs: ferroptosis-inducing agents
Fpn: ferroportin
FTH1: ferritin heavy chain 1
FTL: ferritin light chain
γ-GCS: γ-glutamylcysteine synthetase
GPX4: glutathione peroxidase 4
HCC: hepatocellular carcinoma
4-HNE: 4-hydroxynonenal
HO-1: heme oxygenase 1
HSPB1: heat shock protein family B (small) member 1
IREB2: iron response element binding protein 2
LOXs: lipoxygenases
LPCAT3: lysophosphatidylcholine acyltransferase 3
lysoPC: lysophosphatidylcholine
MDA: malondialdehyde
MT: metallothionein
NCOA4: nuclear receptor coactivator 4
NOXs: NAPDH oxidases
NRF2: nuclear factor, erythroid 2 like 2
PC: phosphatidylcholine
PDAC: prostate adenocarcinoma
PHKG2: phosphorylase kinase catalytic subunit gamma 2
PPP: pentose phosphate pathway
PUFAs: polyunsaturated fatty acids
RCCs: renal cell carcinomas
SLC7A11: solute carrier family 7 member 11
TF: Transferrin
TfR1: transferrin receptor 1
